# Data on arsenic contamination in groundwater of Rafsanjan plain, Iran

**DOI:** 10.1016/j.dib.2020.105772

**Published:** 2020-05-28

**Authors:** Jafar Rahnamarad, Reza Derakhshani, Ahmad Abbasnejad

**Affiliations:** a Department of Geology, Zahedan Branch, Islamic Azad University, Zahedan, Iran; b Department of Geology, Shahid Bahonar University of Kerman, Kerman, Iran; c Department of Earth Sciences, Utrecht University, Utrecht, The Netherlands

**Keywords:** Geogenic arsenic, Groundwater pollution, Rafsanjan plain, Iran

## Abstract

This data article focuses on the arsenic in the groundwater of Rafsanjan plain in Kerman Province of Iran where the groundwater is being extensively used for drinking and irrigation of pistachio gardens. The measured arsenic concentrations range from 4 to 278 μg/L (with an average of 59 μg/L). About 85.3% of water samples have arsenic concentrations above 10 μg/L provided by the World Health Organization, WHO, guideline value. This data article provides also map showing the concentration of arsenic in groundwater of Rafsanjan area based on the situation of the sampling points in Rafsanjan region.

Specifications Table

**Table:** 

**Subject**	Environmental science
**Specific subject area**	Environmental earth science
**Type of data**	Table Figure
**How data were acquired**	Data was acquired from the water samples taken from 41 deep drilled wells driven in Quaternary alluvials (as the aquifer) in Rafsanjan district, and measurement of arsenic in them.
**Data format**	Raw
**Parameters for data collection**	Total inorganic arsenic was determined using inductive-coupled plasma optical emission spectrometry
**Description of data collection**	A total of 41 groundwater samples in Rafsanjan area (Fig. 1) were collected from the drilled wells after 15 min pumping and storage in pre-rinsed low-density polyethylene bottles and then acidified for arsenic analysis by nitric acid. The analytical method was done upon the standard techniques presented by the American Water Works Association, American Public Health Association and Water Environment Federation [1].
**Data source location**	Region: Rafsanjan Country: Iran Latitude and longitude for collected samples: Latitude: 55°,51’ to 56°,19’ N & Longitude: 29°,57’ to 30°,26’ E
**Data accessibility**	With the article

## Value of the data

• This data can be useful for future research on the origin of arsenic as well as for medical geology investigations in this region.
• Considering the presence of volcanic mountains and copper mines in the drainage basin of the region, geologists can benefit this data to interpret the geological formations' influence on arsenic levels of groundwater.
• This data could be compared with the similar ones obtained from the adjacent areas for revealing the arsenic contamination and its possible source.
• This data could be useful for evaluating the role of processes operating on arsenic mobility.
• This data could be useful to detect high-risk areas and explain the cause of arsenic hot spot in this region.

## 1 Data Description

**Fig. 1:**
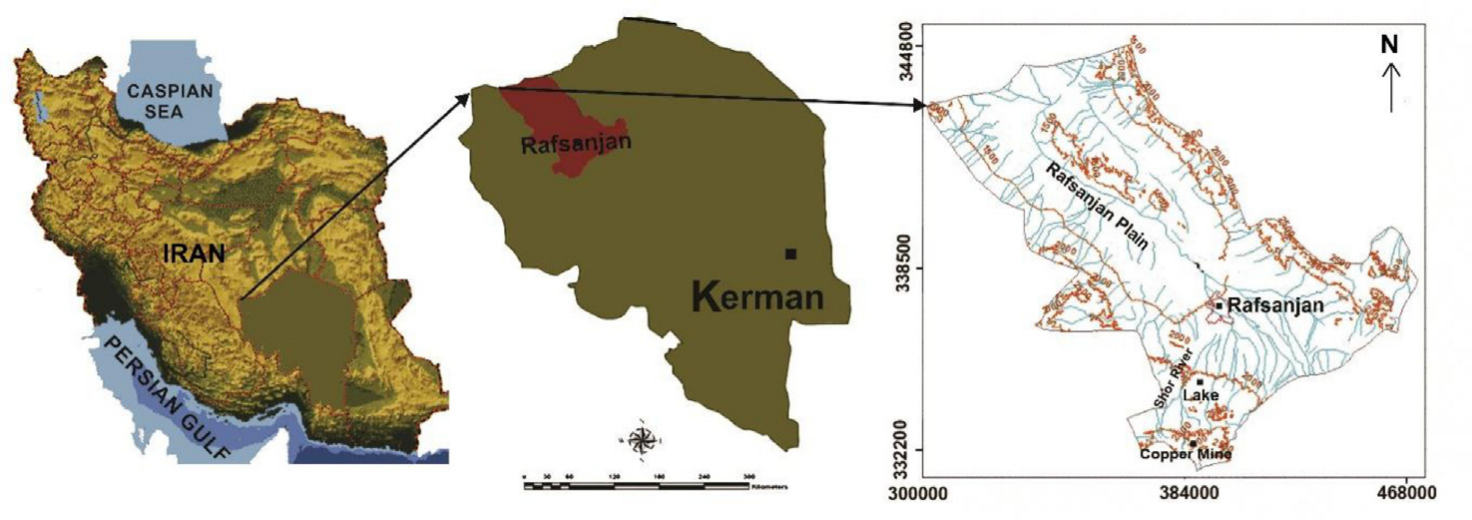
Location of Rafsanjan district in Kerman Province of Iran where the data was acquired.

The presented data describe the concentration of inorganic arsenic in groundwater of Rafsanjan plain in Kerman Province of Iran (Fig. 1). The data is prepared based on fieldwork and laboratory analysis being provided in table and figure form. The main reservoir of groundwater in Rafsanjan district is the Quaternary Alluvials. The main source of replenishment of groundwater in the area is rainfall. The climate of Rafsanjan is hot and arid and its annual rainfall is less than 100 mm [2]. However, precipitation at the high mountains in the south of studied area (the drainage basin of Shour river) reaches up to 300 millimetres per year. Considering the sampling points are mainly located at the downstream parts of Shour river valley and its large alluvial fan, and given the Sarcheshmeh copper mine is located in the drainage basin of this seasonal river, this data could be used for assessing the impact of copper mining on arsenic pollution of groundwater. Irrigation of gardens has been carried out through the qanats in the past [3], but nowadays, due to the severe drop in groundwater level in the plain and subsequent drying of aqueducts, it is practiced by deep wells.

**Fig. 2:**
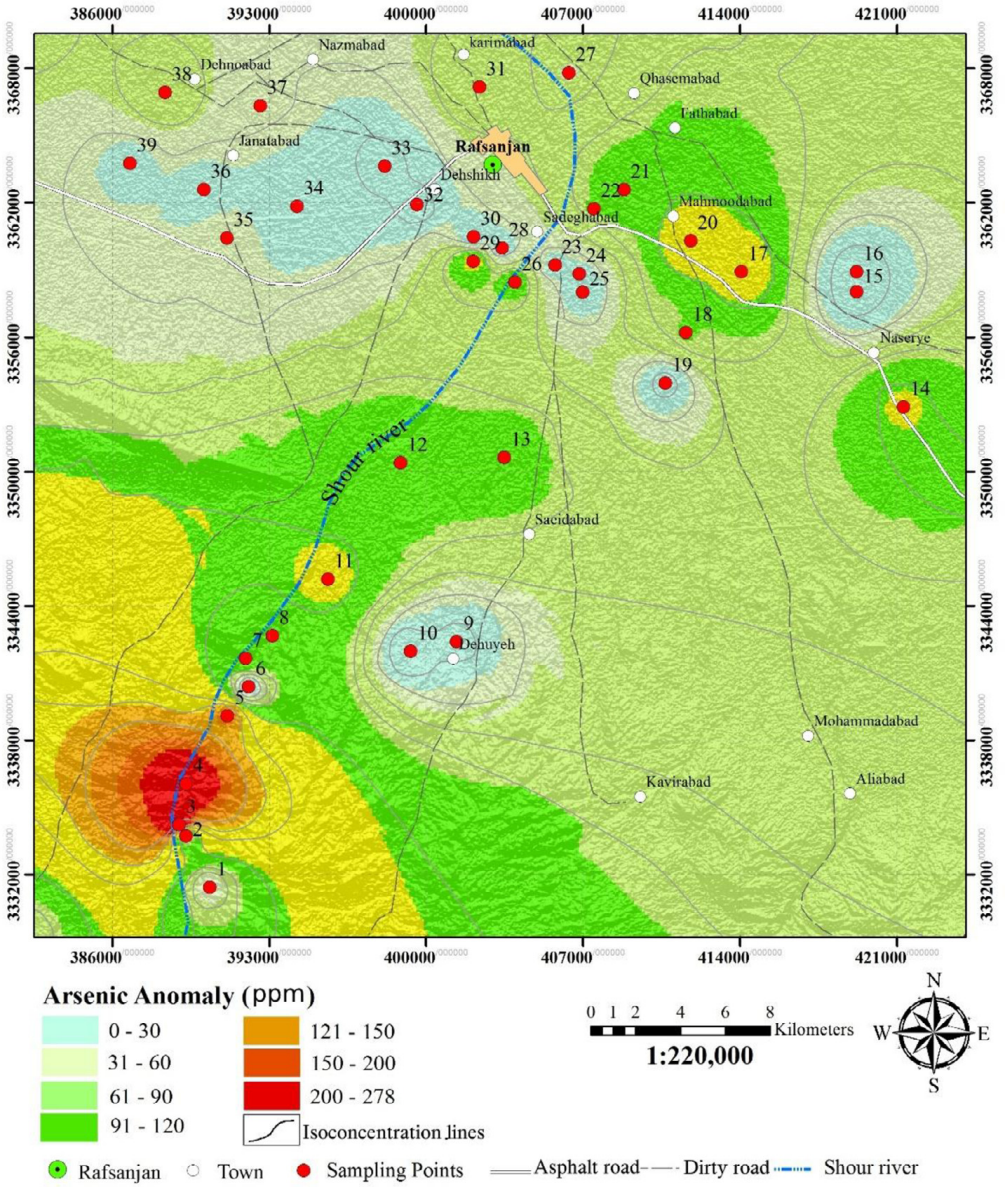
Location of sampling points and arsenic concentration map of groundwaters.

The Shour River which flows from the south creates a large alluvial fan at the south of Rafsanjan plain. This river originates from Sarcheshmeh Mountains and the Sarcheshmeh copper mine is located in the drainage basin. Sarcheshmeh is the largest open pit copper mine in Iran. In Rafsanjan Plain, most of the groundwater wells are being used for irrigation purposes. The location of the sampling points is shown on Fig. 2. Table 1 and Fig. 2 present the amount and concentration of arsenic of groundwater samples of Rafsanjan plain.

**Table 1:** Arsenic concentration in samples (mg/L).

Sample no.	As (mg/l)	Sample no.	As (mg/l)	Sample no.	As (mg/l)
1	20	15	93.9	29	33
2	278	16	8.9	30	13.4
3	259	17	115	31	4.2
4	27.6	18	123	32	152
5	15.2	19	11.7	33	88.7
6	20.1	20	11.1	34	7.5
7	83.9	21	8.9	35	12.7
8	5.2	22	12.9	36	84.9
9	109	23	66.6	37	98.1
10	11.9	24	17.4	38	85.8
11	13.8	25	23.4	39	82.5
12	25	26	31.4	40	40.2
13	66.4	27	23.4	41	59.6
14	83.1	28	59.3		

## 2 Experimental Design, Materials, and Methods

Health problems associated with arsenic pollution in groundwater have been reported in several countries throughout the world, including Bangladesh, Pakistan, India, Nepal, Mexico, China, Hungary, Cambodia, Vietnam, Chile, Argentina, Inner Mongolia, Taiwan, and Egypt [4], [5], [6], [7]. The extent of the problem is so large that millions of people around the world have been affected by arsenic-contaminated waters extracted from aquifers. Long-term consumption of arsenic reach groundwater (above 50 μg/l) has caused endemic arsenic poisoning amongst millions of people. Signs of chronic exposure to arsenic in drinking water commonly include cardiovascular, haematological, neurological, respiratory, renal and skin diseases, as well as liver, lung, bladder, kidney and prostate cancers [8,9].

Evidence for elevated groundwater arsenic contaminations with geogenic source has been increasing over the past 20 years. Commonly, the main sources of arsenic in arsenic-contaminated aquifers are the arsenic-rich minerals (e.g., arsenopyrite, arsenian pyrite, and enargite), mining activity, mine tailings and geothermal waters [7,[10], [11], [12]].

The geological situation of the sampling points is in the Central Iranian Volcanic Zone that is mainly comprised of andesitic–rhyolitic Eocene extrusive and Oligo-Miocene granitoids. Groundwater resources adjacent to this zone are rich in arsenic [10]. Kerman porphyry copper belt that is located at the southern segment of this zone is the main copper ore-rich region in Iran [13], [14], [15], [16], [17].

In this investigation, 41 groundwater samples were taken from deep drilled-wells located across the area (Fig. 2). Samples 1 to 12 are taken from groundwaters in alluvial beds of the downstream parts of Shour River and the others are taken mainly from groundwaters in its alluvial fan. Distribution of wells is not uniform and, consequently, sampling points are not uniformly distributed. Water samples were collected after pumping for at least 15 min and were stored in pre-rinsed 1-litre volume polyethylene bottles. The samples were filtered through a 0.45 µm filter and then acidified by pure nitric acid. The total inorganic acid concentration was determined using inductive-coupled plasma optical emission spectrometry (ICP-OES) (Optima 7000 DV, PerkinElmer, method #3120A).

In order to control the quality of analyses, standard reference material (ERM-CA615, Groundwater-JRC), as well as duplicate and blanks, were used. Quality controls were within ±10% of the certified value. For preparing standard analytical grade solutions, 1000 mg/l of stock solution (Merk, Darmstadt, Germany) was used. Duplicate concentration was within ±10% of relative standard deviation.

## Declaration of Competing Interest

The authors declare that they have no known competing financial interests or personal relationships which have, or could be perceived to have, influenced the work reported in this article.

## Appendix Supplementary materials

Image, application 1
